# Chemical Composition, Analgesic, and Anti-Inflammatory Properties of *Pelargonium odoratissimum* Essential Oils (L.) L’Hérit

**DOI:** 10.3390/ph18101428

**Published:** 2025-09-23

**Authors:** Pamela Rungqu, Opeoluwa Oyedeji

**Affiliations:** Department of Chemistry, Faculty of Science and Agriculture, University of Fort Hare, Alice 5700, South Africa

**Keywords:** *Pelargonium odoratissimum*, essential oils, chemical composition, acute toxicity, analgesic, anti-inflammatory

## Abstract

**Background/Objectives:** *P. odoratissimum* has been traditionally used for its antiseptic and astringent qualities, as well as for treating burns, shingles, ulcers, and wounds. This study sought to explore the chemical profile, analgesic, and anti-inflammatory properties of *P. odoratissimum* essential oils extracted from different parts. **Methods:** The essential oils from fresh and dry *P. odoratissimum* leaves and twigs were extracted using a hydrodistillation method and their chemical profile was elucidated by a GC-MS. In addition, rats were used to test the essential oils’ analgesic effects by submerging the tail in hot water. Paw edema caused by egg albumin was utilized to evaluate the anti-inflammatory impact of the oils; oral dosages of 100, 200 and 400 mg/kg were used in both biological assays. **Results:** The essential oils were found to contain forty-seven different compounds. Among these, the following compounds were some of the oil’s principal compounds: 1.8–4.9% α-selinene, 0.6–5.2% γ-cadinene, 0.6–9.2% γ-selinene, 3.6–10.0% piperitone, 45.7–46.3% *iso*menthone, and 41.8–63.9% menthone. Pharmacological investigation of the essential oils revealed that even the highest dosage of 5000 mg/kg showed no acute toxicity-related deaths. The oils significantly enhanced the time of reaction in analgesic evaluation at dosages of 100, 200, and 400 mg/kg. Moreover, the essential oils also substantially suppressed (*p* < 0.01–0.001) the paw swelling caused by egg albumin at 100, 200, and 400 mg/kg dosages. **Conclusions:** These results confirmed the great potential of *P. odoratissimum* essential oils and their application in traditional medicine.

## 1. Introduction

For millennia, aromatic oils have been utilized for their remarkable capacity to alleviate an extensive range of human ailments, including bronchitis, pneumonia, pharyngitis, diarrhea, hemorrhaging, Hansen’s disease, thermal injuries, sexually transmitted diseases, and mycotic infections. Essential oils are volatile, naturally occurring, intricate blends that include numerous distinctive compounds, mainly monoterpenes, sesquiterpenes, and their related oxygenated compounds. These substances, which have potent aromas and tastes, are secondary metabolites of aromatic plants [1,2]. Each of these components plays a role in the beneficial or adverse effects of essential oils [3]. As valuable natural products, essential oils are extracted from various plant organs like roots, barks, stems, twigs, branches, leaves, buds, flowers, seeds, or fruits. These oils are then used as raw materials in products such as feed additives, flavor enhancers, in cigarettes, and cosmetics [4]. Moreover, these oils are also used in medical fields like pharmacy, therapeutic massage, homeopathic remedies, and hydrotherapy [5,6]. They are excellent natural resources with economic value and serve as promising starting material for chemical synthesis [7]. Indeed, some essential oils seem to demonstrate distinct medicinal attributes that have been suggested as remedies for certain organ dysfunctions or systematic ailments [8]. Since consumers are increasingly aware of the health problems associated with multiple synthetic agents, the use of essential oils as healing therapeutics for health issues is gaining attraction [9].

*P. odoratissimum* (L.) L’Herit. (family Geraniaceae) is an indigenous species from South Africa, prevalent in regions such as KwaZulu-Natal, Eastern and Western Cape, and Mpumalanga provinces in South Africa [10]. This plant has also been noted for its cultivation in Madeira, Colombia, Brazil, Korea, France, Puerto Rico, Spain, Ecuador, and the United States [11]. Commonly referred to as the apple-rose-scented geranium, peppermint pelargonium, or sweet-scented pelargonium. *P. odoratissimum* features very small flowers that are pale pink [12]. The leaves are a vibrant apple green, surrounded by delicate short hairs that provide a delightful tactile experience, emitting a robust apple-mint fragrance. Each leaf spans 30 to 40 mm in length, presenting a rounded to oval heart-shape with scalloped borders. This plant is perennial, growing as a moderately flat shrublet; its primary stem is short and thick, complemented by broad blossoming branches that can reach up to 60 cm in length. The fundamental stem is rough and layered due to the robust foundations of stipules. The roots are somewhat tuberous. *P. odoratissimum* can occasionally reach heights exceeding 30 cm. This plant thrives as an understory in forests and shaded areas shielded by rocky edges and taller bushes [12]. *P. odoratissimum* has various medicinal benefits, serving as a treatment for throat infections, digestive disorders, debility, neuralgia, and hemorrhage. This species of *Pelargonium* is also known for its healing properties for wounds, shingles, burns, and ulcers. The entire plant is recognized as an aromatic herb with tonic, antiseptic, and astringent properties [12,13]. Aqueous leaf extract of *P. odoratissimum* has been found to contain phenolic compounds such as kaempferol, caffeic acid, chlorogenic acid, ferulic acid, cinnamic acid, syringic acid, coumaric acid, naringenin, apigenin, methyl gallate, quercetin, catechin, ellagic acid, and daidzein along with bioactive compounds like saponins, carbohydrates, tannins, glycosides, and flavonoids [14]. Celi et al. [15] reported that methanolic leaf and stalk extracts of *P. odoratissimum* contained flavonoids, epigallocatechin tannins, and gallocatechin. Meanwhile, the hydroalcoholic leaf and stalk extracts contained kaempferol derivatives and quercetin [15]. These bioactive compounds are valued because of their biological activities. Pharmacological investigations using in vivo and in vitro models in *P. odoratissimum* essential oils and extracts have reported them to possess biological properties such as preservative effects on ground beef, antiaflatoxin, insecticidal, antifungal, anti-inflammatory, antioxidant, antibacterial, and diabetes properties [10,14,16,17,18,19]. Additionally, the essential oils extracted from this plant have also been utilized for hormonal regulation, detoxification, and menstrual flow [12,13]. In this context, the purpose of this research was to extract *P. odoratissimum* essential oils from various components, analyze its chemical composition, and assess the analgesic and anti-inflammatory effects of the essential oils.

## 2. Results

### 2.1. Phytochemical Composition of P. odoratissimum Essential Oils

**Figure 1:**
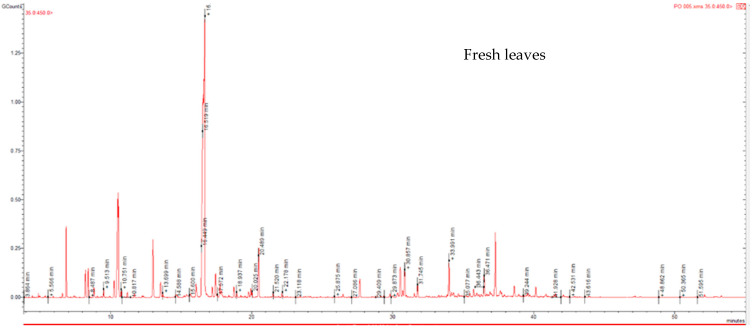

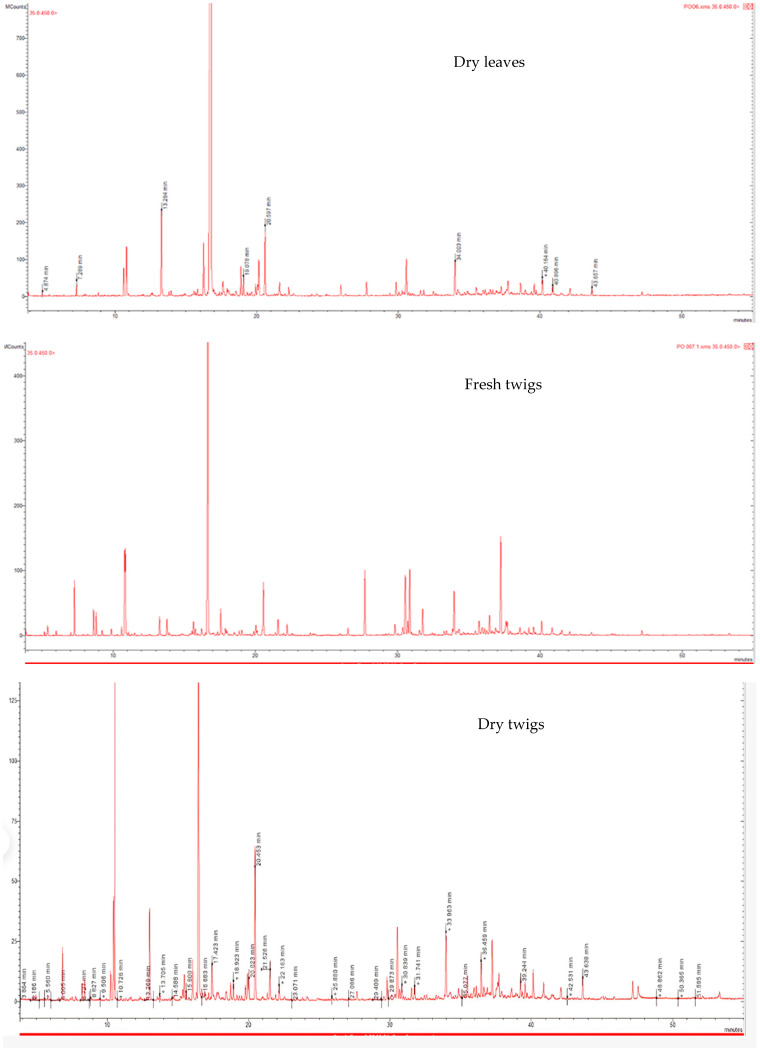
Chromatograms for fresh and dry *P. odoratissimum* leaves and twigs essential oils.

Hydrodistillation of fresh and dry *P. odoratissimum* leaves and twigs yielded essential oils at 3.4% and 2.2% on a fresh weight basis, respectively. While, on a dry weight basis, the essential oils yielded the percentage yields of 3.5% in dry leaves and 2.8% in dry twigs. The compounds identified in both fresh and dry *P. odoratissimum* leaves and twigs essential oils are listed in Table 1, together with their Kovat indices and area %. A total of forty-seven compounds included 47.3–70.2% monoterpenoids, 7.4–17.4% sesquiterpenes, 1.5–11.0% monoterpenes, and 1.9–3.5% sesquiterpenoids. Additionally, non-terpenic chemical groups of compounds were also identified in the essential oils, such as 0.1–21.6% aromatics, 2.6–14.1% saturated hydrocarbons, and 5.2–8.5% others. The analysis using GC-MS revealed the presence of thirty-two, eighteen, eleven, and twelve compounds representing 83.9%, 90.2%, 97.5%, and 93.5% of the total oil content of fresh leaves, dry leaves, fresh twigs, and dry twigs, respectively (Table 1). The essential oils from fresh *P. odoratissimum* leaves were mainly composed of 41.8% menthone, 6.8% *γ*-terpinene, and *α*-gurjunene (6.2%). In contrast, the essential oils from dry leaves were dominated by 63.9% menthone, 4.6% piperitone, and 4.1% β-maaliene. The essential oil from fresh twigs consisted mainly of 46.3% *iso*menthone, 13.0% *p*-xylene, 9.4% 3-methylheptane, 5.3% piperitone, 4.9% *α*-selinene, 4.7% 2,4-dimethylheptane, and 4.3% *o*-xylene as the main representative of the oil. Meanwhile, the essential oil from dry twigs comprised of 45.7% *iso*menthone, 10.0% piperitone, 9.2% *γ*-selinene, 5.2% *γ*-cadinene, and 4.5% crypton as the main compounds. Chromatograms for fresh and dry *P. odoratissimum* leaves and twigs essential oils are displayed in Figure 1.

### 2.2. Acute Toxicity Test

The toxicity levels for fresh and dry *P. odoratissimum* leaves and twigs essential oils were as follows. In the first phase of the trial, no mice died when administered doses of 10, 100, and 1000 were provided. Furthermore, during the second phase of the trial, where doses of 1000, 1600, 2900, and 5000 mg/kg were given, no fatalities were recorded. Observations made 30 min post oral intake of the essential oil and 24 h later showed no changes in the physical state or behavior of the mice. As a result, the estimated oral LD_50_ of *P. odoratissimum* essential oil was determined to exceed 5000 mg/kg p.o.

### 2.3. Analgesic Activity

#### 2.3.1. The Impact of Essential Oil from Fresh *P. odoratissimum* Leaves on Rat’s Tail Immersion in Hot Water

**Figure 2:**
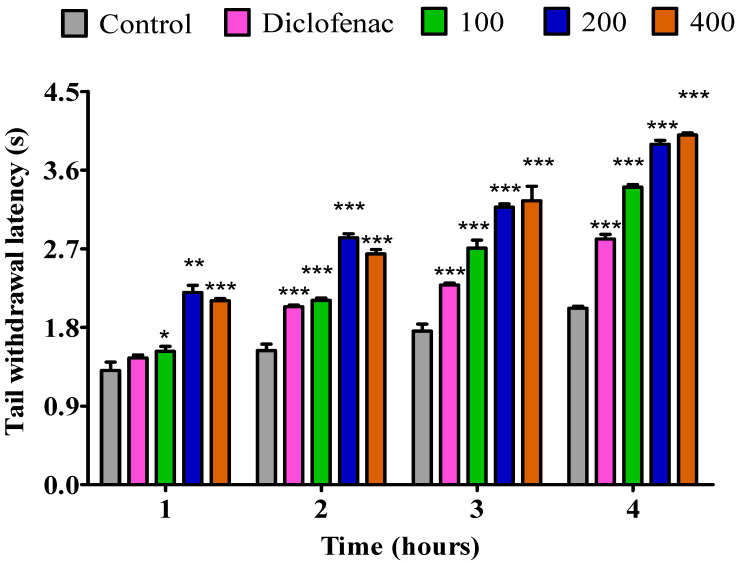
Impact of essential oil from fresh *P. odoratissimum* leaves (F.P.O. L.) on pain induced by heat on rats. Each bar illustrates control (tween 80 and distilled water (10 mg/kg)), diclofenac (100 mg/kg), and F.P.O. L. essential oil (100, 200, and 400 mg/kg), respectively. All treatments were administered orally. * *p* < 0.05, ** *p* < 0.01, and *** *p* < 0.001 indicated significant statistical differences compared to control.

**Figure 3:**
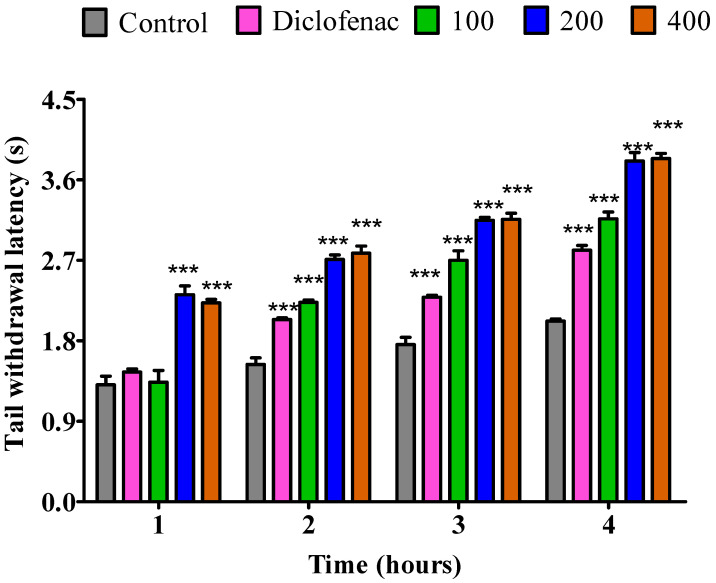
Impact of essential oil from dry *P. odoratissimum* leaves (D.P.O. L.) on pain induced by heat in rats. Each bar illustrates control (tween 80 and distilled water (10 mg/kg)), diclofenac (100 mg/kg), D.P.O. L. essential oil (100, 200, and 400 mg/kg), respectively. All treatments were administered orally. *** *p* < 0.001 indicated significant statistical differences compared to control.

**Figure 4:**
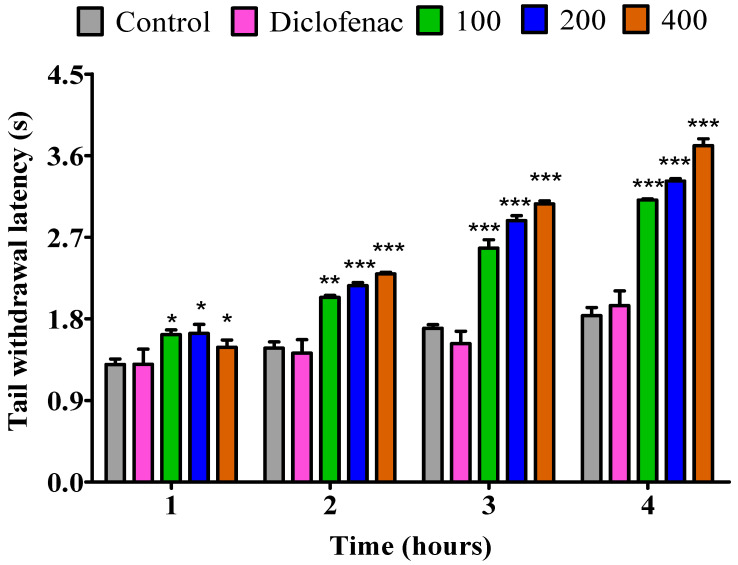
Impact of essential oil from fresh *P. odoratissimum* twigs (F.P.O. T.) on pain induced by heat in rats. Each bar illustrates control (tween 80 and distilled water (10 mg/kg)), diclofenac (100 mg/kg), and F.P.O. T. essential oil (100, 200, and 400 mg/kg), respectively. All treatments were administered orally. * *p* < 0.05, ** *p* < 0.01, *** *p* < 0.001 indicated significant statistical differences compared to control.

**Figure 5:**
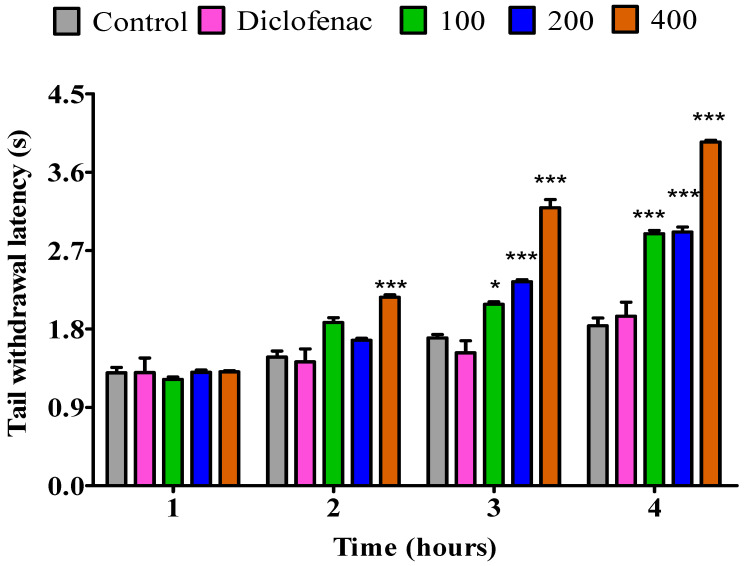
Impact of essential oil from dry *P. odoratissimum* twigs (D.P.O. T.) on pain induced by heat on rats. Each bar illustrates control (tween 80 and distilled water (10 mg/kg)), diclofenac (100 mg/kg), and D.P.O. T. essential oil (100, 200, and 400 mg/kg), respectively. All treatments were administered orally. * *p* < 0.05, *** *p* < 0.001 indicated significant statistical differences compared to the control.

The results of the analgesic effect of *P. odoratissimum* fresh leaves, dry leaves, fresh twigs and dry twigs essential oils are presented in Figure 2, Figure 3, Figure 4 and Figure 5. Thermal stimulus was used to induce pain in rats by submerging the tail of a rat in hot water kept at 55 °C. During the first hour at a dose of 100 mg/kg, the essential oil from fresh leaves exhibited a notable increase (*p* < 0.05) in pain reaction time (PRT) compared to the control. Additionally, during the 1st hour, the essential oil from the fresh leaves exhibited a substantial increase in PRT at dosages of 200 mg/kg (*p* < 0.01) and 400 mg/kg (*p* < 0.001). However, diclofenac did not exhibit any significant increase. Also, compared to the control the essential oil from fresh leaves displayed a significant increase (*p* < 0.001) in PRT throughout the second, third, and fourth hour. When comparing it to diclofenac, the results were comparable (Figure 2).

#### 2.3.2. The Impact of Essential Oil from Dry *P. odoratissimum* Leaves on Rat’s Tail Immersion in Hot Water

The essential oil from dry leaves at dosages of 200 and 400 mg/kg notably prolonged (*p* < 0.001) the PRT during the 1st hour. Conversely, diclofenac and a 100 mg/kg dosage showed no analgesic effect. Moreover, during the second, third, and fourth hour, the 100, 200, and 400 mg/kg dosages exhibited a statistically significant (*p* < 0.001) increase in latency duration compared to the control group. When compared to diclofenac, similar outcomes were recorded (Figure 3).

#### 2.3.3. The Impact of Essential Oil from Fresh *P. odoratissimum* Twigs on Rat’s Tail Immersion in Hot Water

In the first hour, all dosages of 100, 200, and 400 mg/kg of fresh twigs essential oil showed a notable (*p* < 0.05) rise in PRT in comparison to the control group; however, diclofenac exhibited no effect during this period. Furthermore, the essential oil from fresh twigs revealed a significant (*p* < 0.001) increase in PRT analgesic activity throughout the 2nd, 3rd and 4th hour across all doses of 100, 200, and 400 mg/kg in comparison to the control. Diclofenac showed no impact (Figure 4).

#### 2.3.4. The Impact of Essential Oil from Dry *P. odoratissimum* Twigs on Rat’s Tail Immersion in Hot Water

At doses of 100, 200, and 400 mg/kg, the dry twigs showed no significant increase in PRT during the first hour; also, diclofenac showed no analgesic effect. In the second hour, at doses of 100 and 200 mg/kg, no significant increase in PRT was observed in comparison to the control; moreover, diclofenac showed no impact. At a 400 mg/kg dose, dry twigs fundamentally increased the PRT (*p* < 0.001) relative to the control. In the third hour, the essential oil from dry twigs at a dose of 100 mg/kg showed a significant (*p* < 0.05) increase in PRT when compared to control. Conversely, at 200 and 400 mg/kg doses, the essential oil from dry twigs had substantial (*p* < 0.001) analgesic activity in comparison to the control; diclofenac showed no analgesic activity. Additionally, in the fourth hour, dry twigs essential oil exhibited a substantial (*p* < 0.001) increase in all PRT doses of 100, 200, and 400 mg/kg relative to the control. Diclofenac showed no analgesic effect (Figure 5).

### 2.4. Anti-Inflammatory Effect

#### 2.4.1. The Impact of the Essential Oil from Fresh *P. odoratissimum* Leaves on Rat’s Paw Edema Induced by Egg Albumin

**Figure 6:**
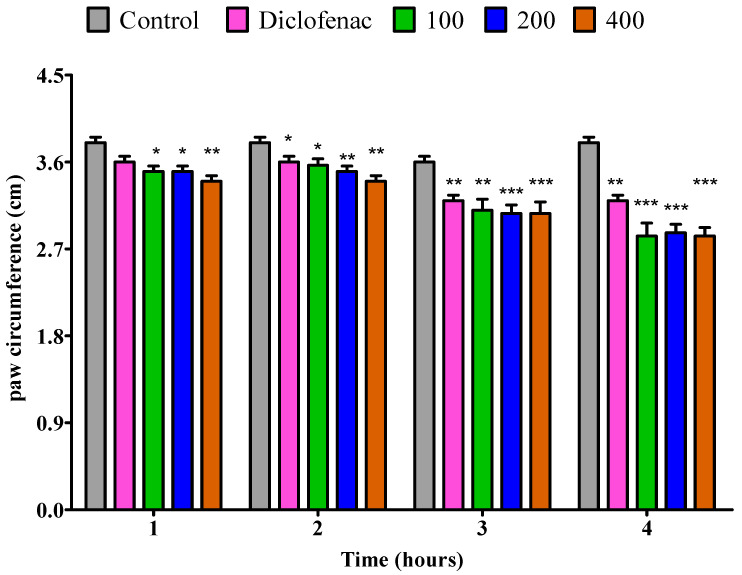
Impact of essential oil from fresh *P. odoratissimum* leaves (F.P.O.L.) on rat’s paw edema induced by egg albumin. Each bar illustrates control (tween 80 and distilled water (10 mg/kg)), diclofenac (100 mg/kg), and F.P.O.L. essential oil (100, 200 and 400 mg/kg), respectively. All treatments were administered orally. * *p* < 0.05, ** *p* < 0.01, and *** *p* < 0.001 indicated significant statistical differences compared to control.

**Figure 7:**
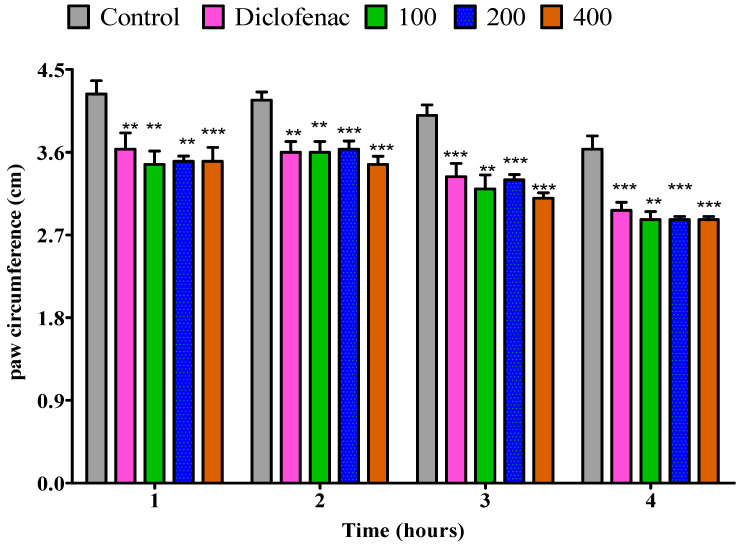
Impact of essential oil from dry *P. odoratissimum* leaves (D.P.O.L.) on rat’s paw edema induced by egg albumin. Each bar illustrates control (tween 80 and distilled water (10 mg/kg)), diclofenac (100 mg/kg), and D.P.O.L. essential oil (100, 200, and 400 mg/kg), respectively. All treatments were administered orally. ** *p* < 0.01, *** *p* < 0.001 indicated significant statistical differences compared to control.

**Figure 8:**
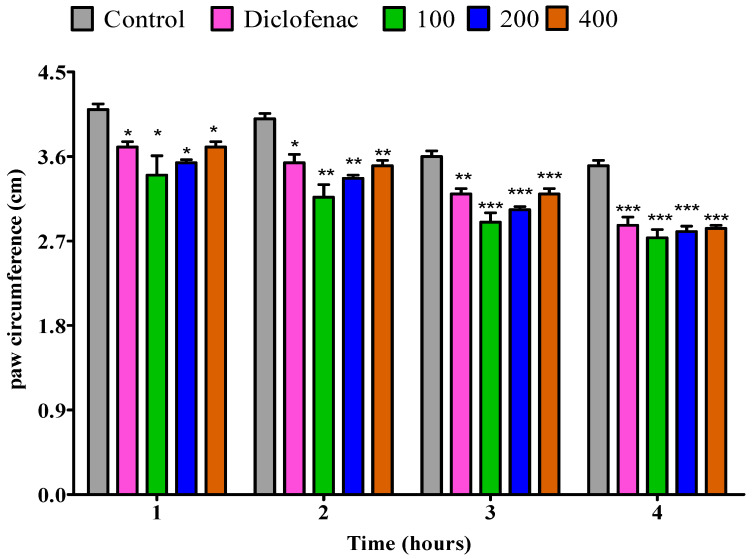
Impact of essential oil from fresh *P. odoratissimum* twigs (F.P.O.T.) on rat’s paw edema induced by egg albumin. Each bar illustrates control (tween 80 and distilled water (10 mg/kg)), diclofenac (100 mg/kg), and F.P.O.T. essential oil (100, 200, and 400 mg/kg), respectively. All treatments were administered orally. * *p* < 0.05, ** *p* < 0.01, *** *p* < 0.001 indicated significant statistical differences compared to control.

**Figure 9:**
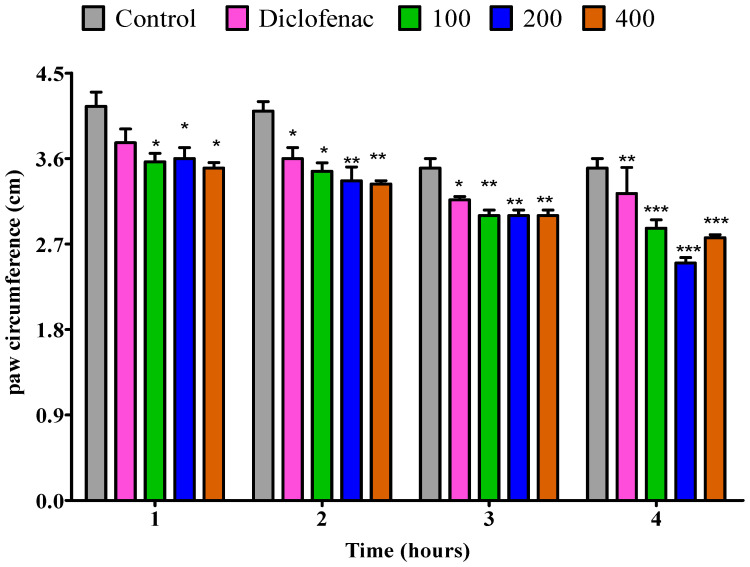
Impact of essential oil from dry *P. odoratissimum* twigs (D.P.O.T.) on rat’s paw edema induced by egg albumin. Each bar illustrates control (tween 80 and distilled water (10 mg/kg)), diclofenac (100 mg/kg), and D.P.O.T. essential oil (100, 200, and 400 mg/kg), respectively. All treatments were administered orally. * *p* < 0.05, ** *p* < 0.01, *** *p* < 0.001 indicated significant statistical differences compared to the control.

The anti-inflammatory activity of *P. odoratissimum*’s essential oils from fresh leaves, dry leaves, fresh twigs, and dry twigs was evaluated by using egg albumin-induced paw edema in the right hind paw of rats; the outcomes of the anti-inflammatory activity are presented in Figure 6, Figure 7, Figure 8 and Figure 9. The essential oil from fresh leaves at doses of 100 and 200 mg/kg exhibited a notable (*p* < 0.05) decrease in the inflamed paw within the first hour. Furthermore, at a dosage of 400 mg/kg, the essential oil significantly (*p* < 0.01) decreased the edema compared to the control. Whereas, when compared to diclofenac, no anti-inflammatory effect was observed. At the 100 mg/kg dose, the essential oil from fresh leaves substantially (*p* < 0.05) reduced the paw volume in egg albumin-induced edema compared to the control throughout the second hour. On the other hand, diclofenac displayed comparable outcomes.In addition, the second hour edema reduction at 200 and 400 mg/kg doses was (*p* < 0.01) in comparison to the control. However, a low anti-inflammatory effect (*p* < 0.05) was observed in comparison to diclofenac. In the third hour, in comparison to the control, essential oil from fresh leaves at a dose of 100 mg/kg notably (*p* < 0.01) inhibited the inflamed paw. Meanwhile, doses of 200 and 400 mg/kg substantially (*p* < 0.001) decreased the paw edema. Conversely, diclofenac displayed a lower inhibition (*p* < 0.01). In addition, all three doses of the fresh leaves essential oil (i.e., 100, 200, and 400 mg/kg) demonstrated an inhibition rate of *p* < 0.001 during the fourth hour in comparison to the control, while diclofenac had a lower (*p* < 0.01) inhibition rate than the 3 fresh leaves essential oil doses (Figure 6).

#### 2.4.2. The Impact of Essential Oil from Dry *P. odoratissimum* Leaves on Rat’s Paw Edema Induced by Egg Albumin

In the essential oil of dry leaves, doses of 100 and 200 mg/kg showed a significant anti-inflammatory activity of (*p* < 0.01) relative control in the first hour; comparable outcomes were noted in comparison to diclofenac. At 400 mg/kg, the dry leaves essential oil exhibited a pronounced anti-inflammatory effect of *p* < 0.001 compared to the control. Furthermore, during the second hour, the 100 mg/kg dose exhibited a noteworthy (*p* < 0.01) reduction in paw edema comparable to the control. Diclofenac demonstrated the same effects. Additionally, the 100 mg/kg dosage exhibited a notable (*p* < 0.01) reduction in the paw edema in comparison to control during the third and fourth. Meanwhile, the 200 and 400 mg/kg doses demonstrated a statistically substantial (*p* < 0.001) decrease in the inflamed paw compared to control, same results were noted when compared to diclofenac (Figure 7).

#### 2.4.3. The Impact of Essential Oil from Fresh *P. odoratissimum* Twigs on Rat’s Paw Edema Induced by Egg Albumin

All three doses of fresh twigs essential oil (i.e., 100, 200, and 400 mg/kg) demonstrated a significant (*p* < 0.05) anti-inflammatory effect comparable to control in the first hour; a similar level of inhibition rate (*p* < 0.05) was noted for diclofenac. Also, a substantial significant (*p* < 0.01) anti-inflammatory effect was observed in the 100, 200, and 400 mg/kg doses in the second hour in comparison to the control. On the other hand, diclofenac had a low inhibition rate (*p* < 0.05). In the third hour, a 100 mg/kg dose of fresh twigs essential oil substantially (*p* < 0.01) decreased the paw edema relative to the control. Moreover, both 200 and 400 mg/kg doses, as well as diclofenac, exhibited an inhibition rate of *p* < 0.001 throughout the third hour. Also, the anti-inflammatory effect of 100, 200, and 400 mg/kg doses was demonstrated by a reduction (*p* < 0.001) in paw volume of rats when compared to the control in the fourth hour. Diclofenac showed similar results (Figure 8).

#### 2.4.4. The Impact of Essential Oil from Dry *P. odoratissimum* Twigs on Rat’s Paw Edema Induced by Egg Albumin

Oral treatment with dry twigs essential oil at 100, 200, and 400 mg/kg doses showed a substantial (*p* < 0.05) reduction in paw edema comparable to control throughout the 1st hour. Diclofenac, however, was inactive. Dry twigs essential oil at 100 mg/kg demonstrated a substantial (*p* < 0.05) inhibition of the inflamed paw in comparison to the control in the second hour. Likewise, the diclofenac displayed similar results. Additionally, 200 and 400 mg/kg doses demonstrated a noteworthy (*p* < 0.01) repression of the inflamed paw as compared to the control. The reductive ability of dry twigs essential oil at 100, 200, and 400 kg/kg doses was found to be (*p* < 0.01) relative to the control throughout the third hour. However, diclofenac exhibited a low (*p* < 0.05) inhibition rate. Furthermore, the anti-inflammatory activity of 100, 200, and 400 mg/kg displayed a significant (*p* < 0.001) decrease in paw volume when compared to the control during the fourth hour. Whereas diclofenac showed a lower anti-inflammatory activity of *p* < 0.01 (Figure 9).

## 3. Discussion

### 3.1. The Chemical Profile of Essential Oils from P. odoratissimum

This study assessed the chemical profile, analgesic, and anti-inflammatory effects of essential oils extracted from fresh and dry *P. odoratissimum* leaves and twigs. To the best of our knowledge, there are no reports on the chemical profile, anti-inflammatory, and analgesic properties of *P. odoratissimum* essential oils from the Eastern Cape in South Africa. Consequently, this research presents the first report on the chemical profile, analgesic, and anti-inflammatory properties of *P. odoratissimum* essential oils sourced from the Eastern Cape region of South Africa. Previous studies have reported the chemical composition of *P. odoratissimum* essential oils, revealing significant variations, especially from those obtained from different countries. This investigation is different from the *P. odoratissimum* essential oils found in Poland, where the main compounds identified in the essential oil were 2.23% menthone, 2.45% linalool, 3.34% *iso*menthone, 6.51% geraniol, 8.08% *allo*-aromadendrene, 9.22% citronellyl formate, and 29.77% citronellol [24]. In Colombia, the predominant compounds identified in the essential oil of *P. odoratissimum* leaves comprised of 3.32% cinnamyl acetate, 4.18% phenyl-ethyl-tiglate, 4.55% *cis*-α-bisabolene, 5.74% phenyl-ethyl-butanoate, 8.99% citronellol, and 12.69% geraniol [25].

Khalid et al. [26] analyzed the essential oil from the seedlings of *P. odoratissimum* sourced from Shanghai, China, using three extraction techniques: hydrodistillation (HD), hydro-steam distillation (HD-SD), and steam distillation (SD). The authors reported high contents of 26.5–26.7% *iso*menthone, and 25.9–30.4% methyl eugenol in HD, while HD-SD featured 16.2–21.3% methyl eugenol, 19.2–20.8% *iso*menthone and 30.5–32.4% limonene as the predominant compounds. Conversely, in SD, 12.2–14.4% methyl eugenol, 15.1–19.3% *iso*methone, and 38.8–49.4% limonene were found to be the main compounds. In contrast, the leaves and flowers of *P. odoratissimum* essential oil from Italy, as analyzed by Ben Hsouna and co-authors [10], contained 2.6% geranyl acetate, 4.8% linalool, 7.4% *iso*menthone, 9.8% (-)-aristolene, 12.6% citronellyl formate, 15.3% nerol, and 40.0% citronellol as the major compounds. In another study, Benelli *et al*. [27] analyzed the commercial essential oil of *P. odoratissimum* from Italy, revealing 5.7% 6,9-guaiediene, 9.1% citronellyl formate, 16.2% *iso*menthone, and 30.1% citronellol as the most abundant compounds. Matusinsky et al. [17] investigated the commercial essential oil of *P. odoratissimum* from the Czech Republic, reporting 4.73% linalool, 5.95% *γ*-eudesmol, 6.19% *iso*menthone, 7.72% citronellyl formate, 12.50% geraniol, and 24.86% *β*-citronellol as the main compounds of the essential oil.

A comparative analysis of the essential oils extracted from *P. odoratissimum* located in the Eastern Cape region of South Africa, when contrasted with those previously studied from various countries of the world, indicated a significant variation in their chemical composition. Nonetheless, several similarities were noteworthy. This investigation identified menthone, *iso*menthone, *p*-xylene, piperitone, 3-methylheptane, *γ*-selinene, *γ*-terpinene, *α*-gurjunene, *γ*-cadinene, *α*-selinene, 2,4-dimethylheptane, crypton, *o*-xylene, and *β*-maaliene as the primary compounds of *P. odoratissimum* essential oil. Contrary to this observation, the essential oils extracted from the leaves of *P. odoratissimum* in Poland [24] identified 8.08% *allo*-aromadendrene as the predominant compound, whereas it was only detected in low amounts (1.7%) in our study. Furthermore, the essential oils contained 3.34% menthone and 2.23% *iso*menthone at lower levels, while our investigation revealed 41.6–63.9% menthone and 45.7–46.3% *iso*menthone at significantly high concentrations. Notably, the main compounds identified in the essential oil, such as citronellol, citronellyl formate, geraniol, and linalool, were not found in our results. The primary compounds identified in Colombian [25] *P. odoratissimum* essential oil leaves, including ethyl-phenyl-tiglate, cinnamyl acetate, geraniol, phenyl-ethyl butanoate, citronellol, and *cis*-*α*-bisabolene, were absent from this study.

In addition, the commercial essential oils of *P. odoratissimum* obtained in the Czech Republic contained geraniol, *iso*methone, linalool, citronellyl formate, and *γ*-eudesmol [17] as the main compounds. Meanwhile, in Italy [24], the primary compounds identified in the commercial essential oil of *P. odoratissimum* were citronellol, *iso*menthone, citronellyl formate, and 6,9-guaiediene. Our research, however, did not detect citronellol, geraniol, citronellyl formate, linalool, 6,9-guaiediene, and *γ*-eudesmol. Nevertheless, *iso*menthone was available at relatively higher amounts (45.7–46.3%) compared to that reported in Italy (16.2%) [27] and the Czech Republic 6.19% [17]. Ben Hsouna *et al*. [10] reported that the essential oils extracted from leaves and flowers of *P. odoratissimum* in Italy contained citronellol, nerol, citronellyl formate, (-)-aristolene, *iso*menthone, linalool, and geranyl acetate as the main representatives of the essential oil. While *iso*menthone was also found in our *P. odoratissimum* essential oil, we did not detect citronellol, nerol, citronellyl formate, (-)-aristolene, linalool, and geranyl acetate in the essential oil. The primary compounds found in the essential oil seedling of *P. odoratissimum* extracted from Shanghai, China [26], through different extraction methods predominantly contained methyl eugenol, *iso*menthone, and eugenol in the HD method. The HD-SD method showed that the essential oils comprised of limonene, *iso*menthone, and methyl eugenol. On the other hand, the SD method revealed that limonene, *iso*methone, and methyl eugenol were the primary compounds of the oil. However, our investigation did not detect methyl eugenol, limonene, and eugenol. In contrast, *iso*menthone, however, was detected in larger quantities (45.7–46.3%) compared to the *iso*menthone levels reported in Shanghai, China, which were 15.1–26.9%.

It is important to note that the *P. odoratissimum* essential oil reported in Poland, Colombia, the United Kingdom, Brazil, China, Italy, and the Czech Republic contained a distinct chemical composition compared to those obtained from this research. The time of plant collection, climate and humidity, geographic location, extraction technique, and other environmental changes may all contribute to the variances in chemical composition of the same species [28].

### 3.2. Acute Toxicity and Analgesic Effect of P. odoratissimum Essential Oils

The current research examined the acute toxicity, analgesic, and anti-inflammatory properties of essential oils extracted from fresh and dry *P. odoratissimum* leaves and twigs. In the oral administration of the essential oils from various parts of *P. odoratissimum*, no signs of toxicity or death occurred, even at the highest dose of 5000 mg/kg of the oils. Consequently, the acute toxicity results for *P. odoratissimum* essential oils demonstrated that the LD_50_ is greater than or equal to 5000 mg/kg (LD_50_ ≥ 5000), implying that the essential oils of *P. odoratissimum* are either nontoxic or possess a very low toxicity profile.

The essential oils extracted from fresh and dry *P. odoratissimum* leaves and twigs at 100, 200, and 400 mg/kg doses were assessed for their analgesic and anti-inflammatory effects in experimental animal models. The analgesic effects of the essential oils were evaluated using a tail immersion test in hot water, while the anti-inflammatory properties were assessed by inducing paw edema with egg albumin, with diclofenac serving as the reference drug. Both fresh and dry leaves essential oils demonstrated significant analgesic effects (*p* < 0.05–0.001) at all tested doses throughout the 4 h duration. Additionally, orally administered essential oils from both fresh and dry twigs prolonged the response latency to heat stimulus across all the doses from the first to the fourth. Notably, the most significant reduction in paw edema was recorded during the second, third, and fourth hour at doses of 200 and 400 mg/kg (*p* < 0.001). The analgesic properties of the essential oils obtained from *P. odoratissimum* fresh leaves, dry leaves, fresh twigs, and dry twigs may result from a combined impact involving the predominant compounds, including *γ*-terpinene, *iso*menthone, and menthone.

Certainly, previous studies have indicated that the levels of 2.73–15.74% and 1.81–7.22% menthone and 2.03–7.73% and 0.82–3.32% *iso*menthone in the essential oils of *Mentha piperita pallescens* and *M. spicata*, among the major compounds of the essential oils, might play a role in the antinociceptive effects of the essential oils. When evaluating the essential oils from the two *Mentha* species (125, 200, and 500 mg/kg) in animal models of nociception, *M. spicata* essential oil exhibited substantial dose-dependent antinociceptive properties by reducing the incidence of writhing across all administered doses. Furthermore, the essential oils of *M. piperita pallescen*s showed a remarkable antinociceptive response in the animal experimental model [29]. In a study conducted by Pina and colleagues [30] on the anti-hyperalgesia impact of *γ*-terpinene (γ-TPN) using a tumor cell-induced neuropathic pain model. The authors found that the mechanical hyperalgesia caused by the introduction of 2 × 10^6^ sarcoma cells 180 in the area surrounding the sciatic nerve was significantly reduced (*p* < 0.001) after 6 days of daily treatment with *γ*-TPN (50 mg/kg, p.o.). In addition, Silva et al. [31] investigated the antinociceptive activity of *γ*-terpinene (GT) using a carrageenan-induced paw edema model to assess edema and mechanical hyperalgesia, with evaluations conducted through Von Frey tests and plethysmometry. According to their findings, GT showed a dose-dependent decrease in mechanical hyperalgesia and paw edema, indicating its analgesic effects.

The analgesic effect of fresh and dry *P. odoratissimum* leaves and twigs essential oils may be mediated by peripheral suppression of prostaglandins synthesis or action [32] and also attributed to the synergistic interaction between the main compounds [33].

### 3.3. Anti-Inflammatory Activity of P. odoratissimum Essential Oils

The research results regarding the anti-inflammatory properties of fresh and dry *P. odoratissimum* leaves and twigs essential oils displayed a substantial (*p* < 0.05–0.001) repression in paw size throughout the 4 h.

The observed anti-inflammatory properties of the essential oils are likely attributed to compounds such as menthone, *iso*menthone, piperitone, and *γ*-terpinene, which are among the principal compounds identified in the essential oils of this study. Research by Golestaneh Talaei et al. [34] noted the anti-inflammatory activity of *Cymbopogon schoenanthus* essential oil at doses of 50, 100, and 200 mg/kg, i.p., in a rat model through carrageenan-induced paw edema studies. The primary compound present in the essential oil was 62.0% piperitone. The researchers found that *C. schoenanthus* essential oils, across all three dosages, resulted in a marked decrease in paw edema caused by carrageenan. In addition, research conducted by Messaoudi and colleagues [35] assessed the anti-inflammatory properties of *Mentha pulegium* essential oil on mice paw edema induced by carrageenan. The chemical profile of the essential oils revealed that 13.01% menthone and 3.82% piperitone were among the prominent compounds of the essential oil. Upon testing the essential oil for its ant-inflammatory effects, it notably reduced the carrageenan-induced paw edema. Furthermore, Bai and co-authors [36] investigated the potential of *Schizonepeta tenuifolia* essential oil using nitric oxide (NO) inhibitory essays. In their anti-inflammatory effect evaluation, the authors included 13 peaks (inclusive of *iso*menthone, piperitone, and menthone) identified from *S. tenuifolia* and essential oils, as well as its essential oil. The findings showed that menthone was thought to be the main anti-inflammatory component; however, *iso*menthone, and piperitone had a notable impact on anti-inflammatory activity.

Similarly, Su and Li [37] used mouse lung mast cells to investigate the anti-allergic inflammatory potential of five terpenoid compounds: menthone, farnesol, oridonin, *β*-escin, and lupeol. Out of the five terpenoid compounds that were chosen, only menthone treatment reduced the ratios of Tumor Necrosis Factor-α/Interleukin-10 (TNFα/IL-10) release in lipopolysaccharide-stimulated mast cells. The researchers further assessed menthone’s ability to treat ovalbumin (OVA)-sensitized and challenged BALB/c mice by gavage for five weeks. The findings showed that five weeks of menthone supplementation restored the percentage of monocytes/macrophages in the lungs while inhibiting eosinophilia, mast cell degranulation, and the expression levels of the genes for CXC receptor 1 and CC receptor 3. In a study conducted by Tan et al. [38], the *γ*-terpinene (GL6a) anti-inflammatory effects were assessed on inflamed T-helper 17 (Th17) and regulatory T (Tregs) cells; the reported results revealed that Tumor Necrosis Factor (TNF), Nuclear Factor Kappa B-1 (NFKB1), retinoic-acid receptor C (RORC), Forkhead Box P3 (FOXP3), cannabinoid receptor-1 (CNR1), interleukin-6 (IL6), IL10, transient receptor potential vanilloid-1 (TRPV1), and 5-hydroxytryptamine receptor 1A. (HTR1A) gene expression levels were all markedly decreased by *γ*-terpinene. Furthermore, Silva et al. [31] explored the anti-inflammatory properties of *γ*-terpinene (GT) in acute joint inflammation. An intra-articular injection of zymosan was used to generate joint inflammation in order to measure myeloperoxidase (MPO) enzyme activity, leukocyte migration, and joint edema. The findings demonstrated that GT had an anti-inflammatory impact by significantly reducing leukocyte migration, joint edema, and MPO activity in the zymosan model. The current results obtained have shown that both fresh and dry *P. odoratissimum* leaves and twigs essential oils possess anti-inflammatory properties. Consequently, the anti-inflammatory action observed may relate to the essential oils’ capacity to inhibit the inflammation mediators such as histamine, leukotrienes, and prostaglandins from both the first and second phases.

Although some achievements were attained. The study has some limitations which include the use of egg albumin instead of carrageenan. While both models have their advantages, using egg albumin instead of carrageenan may present certain limitations. One of the primary limitations of using egg albumin lies in the unique inflammatory reaction it triggers. Egg albumin is often used to study allergic or immune-mediated inflammation, which may not be directly comparable to the acute inflammation induced by carrageenan. This difference inflammatory response may affect the study’s outcomes and limit the applicability of the findings to different forms of inflammation. Another limitation is to only assess the biological effects of *P. odoratissimum* essential oils, without also exploring the primary compounds identified in *P. odoratissimum* essential oils.

Essential oils are complex mixture of various compounds, each possessing its unique chemical structure and biological activity. Therefore, without isolation and characterization the major compounds in essential oils, it becomes difficult to attribute their biological effects to specific compounds. Furthermore, interactions between compounds in essential oils may result in synergistic effects that are not fully understood without studying individual compounds. Future studies should aim to conduct comparative studies using both egg albumin and carrageenan models. This approach would provide a more comprehensive understanding of the anti-inflammatory effects of the essential oils and their potential therapeutic applications. Moreover, isolation and characterization of the major compounds in essential oils, would enhance understanding of their biological effects and enable the development of more targeted therapeutic strategies.

## 4. Materials and Methods

### 4.1. Collection of P. odoratissimum and Authentication

**Figure 10:**
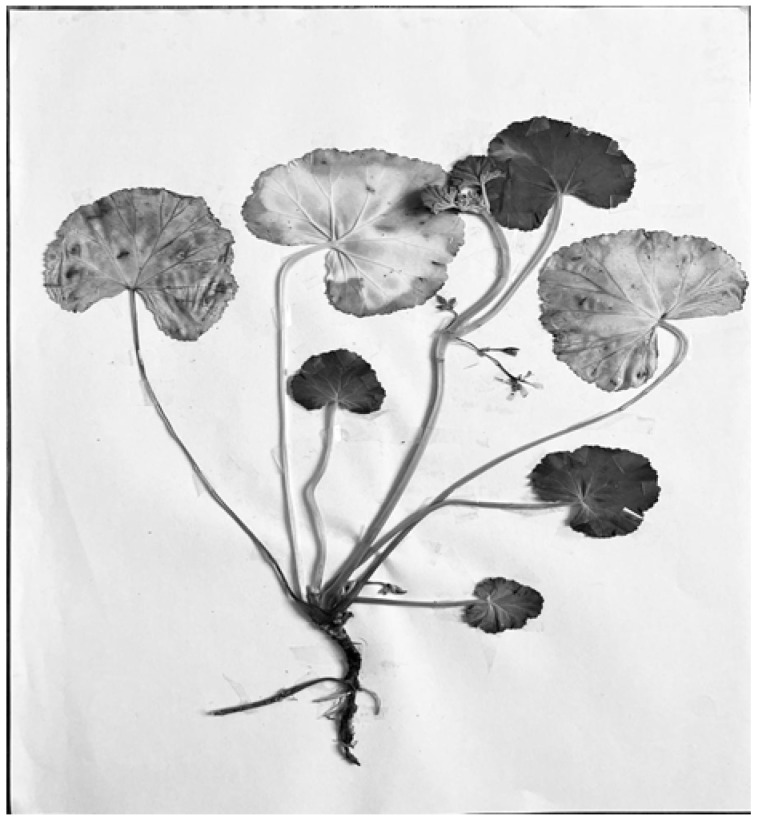
*P. odoratissimum* voucher specimen.

*P. odoratissimum* plant sample was collected along the R67 provincial road between Makhanda (Grahamstown) and Fort Beaufort in the Eastern Cape, South Africa, in March 2017, at the geographical coordinates 26°37′0.85″ N, 33°05′0.85″ W. A voucher specimen (Figure 10) (PR/PL01) was deposited and authenticated by T. Dold at Selmar Schonland Herbarium (GRA), Rhodes University, South Africa.

### 4.2. Essential Oil Extraction

Fresh and dry leaves and twigs of *P. odoratissimum* essential oils were obtained by hydro-distillation for 5 h using the Clevenger apparatus; the parts of the plant material were extracted separately. Fresh leaves (600 g) were placed in a round-bottom flask, and distilled water was added to the bottom flask until it submerged the plant material. The flask was then set within a heating mantle, heated to boil at 100° C, and subsequently reduced to 70 °C. The same procedure was applied to dry leaves, along with fresh and dry twigs. The essential oil was collected and then stored in sealed amber bottles, which were then refrigerated at 4 °C until subjected to analysis and biological studies [39]. All the chemicals and reagents were purchased from Sigma-Aldrich Chemical Co.’s outlet, Shalom Laboratory Suppliers, located in St. Louis, MO, USA.

### 4.3. Essential Oil Analysis

GC-MS analysis of fresh and dry (leaves and twigs) essential oils from *P. odoratissimum* was carried out on a [Bruker 450 gas chromatography, Billerica, Massachussets, USA] coupled to a 300/MS/MS mass spectrometer system running in EI mode at 70 eV, outfitted with an HP-5 MS fused silica capillary system with 5% phenylmethylsiloxane stationary phase. With a film thickness of 0.25 µm, the capillary column’s parameters were 30 m × 0.25 m. At a rate of 5 °C per minute, the column’s initial temperature of 50 °C was raised to 240 °C, and it was maintained at 300 °C for a run time of 66.25. At a flow rate of 1.0 mL per minute, helium was employed as a carrier gas. 100:1 was the split ratio. The scanning range was 35 to 450 amu, and the scan time was 78 min. For analysis, a single microliter (1 µL) of the diluted oil (in hexane) was injected. The same Kovats indices were used to run the N-alkane from C_8_ to C_30_. GC indices obtained from our group library and those found in the literature [20,21,22,23] were used to identify the constituents. Under these operating conditions, the retention indicators were determined in reference to a homologous series of alkanes.

### 4.4. Essential Oil Identification

Identification of compounds was accomplished by comparing retention indices and mass spectra to those recorded in the NIST08 library, as well as by comparing retention indices and mass spectra to literature values [20,21,22,23].

### 4.5. Bioassays

#### 4.5.1. Experimental Animals

Male Wistar albino rats weighing 200–300 g and male Swiss mice weighing 30–40 g were housed in standard cages, with six rats per cage. The temperature was kept at 24 °C, and either electronic or natural light was employed for lighting. The animals were provided with standard rodent pellet diet and water. 8 h before experimentation the animals were deprived of food; however, had free access to drinking water. The essential oils were emulsified with tween 80 (5%) in this study before administration to the animals. Walter Sisulu Faculty of Health Sciences Ethics committee gave ethical clearance for the study (ethical clearance certificate number. 003A/2018). The experimental animals were acquired from the South African Vaccine Initiative (SAVI), located in Johannesburg South Africa.

#### 4.5.2. Drug Used

South Africa’s Reckitt Benckiser Pharmaceutical (PTY) LTD/EDMS BPK provided the diclofenac.

#### 4.5.3. Toxicity Evaluation

The investigation of toxicity to estimate the safety of *P. odoratissimum* essential fresh or dry (leaves or twigs) essential oils was performed on Swiss mice [40]. The study was divided into two phases. In the first phase, nine mice were randomly divided into three groups of three mice each. Group I received a dose of 10 mg/kg of fresh or dry (leaves or twigs) essential oil, while group II and group III received doses of 100 and 1000 mg/kg of fresh or dry (leaves or twigs) essential oil, respectively. The mice were observed for signs and symptoms of toxicity and mortality for 24 h after treatment. In the second group, four mice were divided into four groups of one mouse each. Group I received a fresh or dry (leaves or twigs) essential oil dose of 1000 mg/kg, group II received a fresh or dry (leaves or twigs) essential oil dose of 1600 mg/kg, and group III and group IV received the doses of 2900 and 5000 mg/kg of fresh or dry (leaves or twigs) essential oil, respectively. The mice were monitored for 24 h afterward for deadly effects that lead to death. LD_50_ for fresh and dry *P. odoratissimum* leaves or twigs essential oil was estimated as the geometric mean of the lowest dose causing death and the highest dose causing no death according to Equation (1) below.

(1) $${LD}_{50}=\sqrt{A\times B}$$

where A is the maximum dose that results in 0% death and B is the dose that results in 100% death [40]. The working dosages were calculated using the following Equation (2) based on the LD_50_ result.

Working doses ≤ ½ (LD_50_) (2)

#### 4.5.4. Analgesic Effect: Test of Tail Immersion

The rats were randomly distributed to groups of 14 with 6 rats each. The animals were fasted for 12 h with free access to clean water for drinking before drug administration, and then they were pretreated 60 min before tail immersion in hot water [41]. The treatment groups were treated as follows:

Group I served as a control and was given 5% tween 80, 10 mg/kg; group II was given 100 mg/kg diclofenac; and groups III–V were given fresh *P. odoratissimum* leaves (F.P.O. L) essential oil (100, 200, and 400 mg/kg), respectively. To evaluate the analgesic activity of dry *P. odoratissimum* leaves (D.P.O. L) essential oil, fresh *P. odoratissimum* twigs (F.P.O. T) essential oil, and dry *P. odoratissimum* twigs (D.P.O. T) essential oil, a similar experimental design was carried out, and the experiment for the different *P. odoratissimum* essential oils was performed on different days. After marking the three to four sections, it was then placed in a beaker with hot water in which the temperature was kept at ±55 °C. The pain reaction time (PRT), also referred to as tail flick latency, was recorded as the time it took the rat’s tail to flick or withdraw from the water. The reaction time was measured prior to and 1 h, 2 h, 3 h, and 4 h after the administration of the respective treatments.

#### 4.5.5. Impact of *P. odoratissimum* Essential Oils on Rat’s Paw Induced by Egg Albumin

In this work, the anti-inflammatory properties of *P. odoratissimum* essential oils were investigated in male Wistar rats. The rats received the following treatment after being split up into 14 groups of six rats each:

Group I received 5% tween 80, 10 mg/kg (control); group II received 100 mg/kg diclofenac. Groups III, IV, and V received fresh *P. odoratissimum* leaves (F.P.O. L) essential oil (100, 200, and 400 mg/kg, respectively); groups VI, VII, and VIII received dry *P. odoratissimum* leaves (D.P.O. L) essential oil (100, 200, and 400 mg/kg, respectively); groups IX, X, and XI received fresh *P. odoratissimum* twigs (F.P.O. T) essential oil (100, 200, and 400 mg/kg); and groups XII, XIII, and XIV received dry *P. odoratissimum* twigs (D.P.O. T) essential oil (100, 200, and 400 mg/kg, respectively).

To ensure consistent hydration, rats were fasted for 12 h before the experiment, and only pure tap water was offered for drinking. This was followed by intraperitoneal administration of the test substances as described above. Acute inflammation was induced 30 min after the test substances were administered by injecting 1 milliliter of fresh egg albumin at 50% (*v*/*v*) into the right hind paw’s subplantar region. The increase in the size of each rat’s right hind paw was then measured at 1, 2, 3, and 4 h after egg albumin injection; baseline paw measurements were taken with vernier calipers [42].

#### 4.5.6. Statistical Analysis

The difference between the control groups and treatment groups was statistically analyzed by one-way analysis of variance (ANOVA) followed by Dunnett’s test; *p* < 0.05 was considered statistically significant. Results were expressed as mean ± standard error mean (SEM). Analysis of the results was performed through the GraphPad Prism 5.0 software (GraphPad Software, La Jolla, CA, USA).

## 5. Conclusions

This research marks the first study on the chemical profile, analgesic, and anti-inflammatory properties of essential oils extracted from the fresh and dry *P. odoratissimum* leaves and twigs sourced in the Eastern Cape of South Africa. A total of thirty-two, eighteen, eleven, and twelve compounds were detected in the fresh leaves, dry leaves, fresh twigs, and dry twigs, respectively. Even at the maximum dosage of 5000 mg/kg, the essential oils’ toxicity evaluation did not result in any fatalities. Furthermore, the essential oils derived from fresh and dry *P. odoratissimum* leaves and twigs demonstrated an enhancement in pain reaction time during the analgesic testing in rats while also mitigating the paw edema caused by egg albumin across both phases of inflammation. These findings support the use of *P. odoratissimum* in traditional medicine by indicating that the essential oils from its various sections have analgesic and anti-inflammatory properties. Thus, the essential oils of this plant may present potential as effective pain-relieving and inflammation-reducing agents.

## Figures and Tables

**Table 1:** Chemical composition of the essential oils extracted from fresh and dry *P. odoratissimum* leaves and twigs using hydrodistillation method.

No.	KI^a^	KI^b^	Compounds	Area (%)			
				Fresh Leaves	Dry Leaves	Fresh Twigs	Dry Twigs
1	762	763	Toluene	-	-	2.3	-
2	767	793	3-Methylheptane	-	-	9.4	-
3	822	823	2,4-Dimethylheptane	-	-	4.7	2.6
4	866	907	*m*-Xylene	-	-	2.0	-
5	883	888	*p*-Xylene	-	-	13.0	2.6
6	896	907	*o*-Xylene	-	-	4.3	-
7	939	939	*α*-Pinene	0.2	-	-	-
8	976	969	Sabinene	-	1.5	-	3.2
9	980	978	*β*-Pinene	0.2	-	-	-
10	1005	1007	*α*-Phellandrene	0.3	-	-	-
11	1026	1028	p-Cymene	-	0.5	-	-
12	1031	1030	*β*-Phellandrene	0.6	-	2.8	-
13	1040	1043	*cis*-*β*-ocimene	0.3	-	-	-
14	1053	1052	*trans*-*β*-ocimene	0.5	-	-	-
15	1062	1060	*γ*-Terpinene	6.8	-	-	-
16	1154	1153	Menthone	41.8	63.9	-	-
17	1164	1162	*iso*Menthone	-	-	46.3	45.7
18	1177	1174	Terpinen-4-ol	0.9	-	-	-
19	1189	1186	Crypton	2.2	2.3	-	4.5
20	1193	1195	Myrtenal	0.4	0.5	-	-
21	1210	1210	Pseudocumohyroquinone	0.2	-	-	-
22	1229	1225	*cis*-Carveol	-	0.5	-	-
23	1239	1237	Cuminaldehyde	1.4	1.9	-	2.0
24	1251	1249	Piperitone	3.6	4.6	5.3	10.0
25	1287	1288	*p*-Cymen-7-ol	0.6	0.7	-	-
26	1375	1379	*β*-Elemene	0.3	-	-	-
27	1380	1388	*β*-Maaliene	-	4.1	-	-
28	1380	1379	*β*-Patchoulene	1.3	-	-	-
29	1387	1405	*iso*Longifolene	0.7	-	-	-
30	1402	1405	Longifolene	0.7	0.8	-	-
31	1409	1410	*α*-Gurjunene	6.2	-	-	-
32	1461	1460	*Allo*-aromadendrene	1.7	-	-	-
33	1480	1483	Germacrene D	-	0.9	2.5	-
34	1484	1486	*γ*-Selinene	0.6	-	-	9.2
35	1485	1484	α-Armophene	0.4	0.5	-	-
36	1494	1498	α-Selinene	1.8	2.5	4.9	3.0
37	1499	1500	α-Muurolene	-	1.3	-	-
38	1505	1507	α-Bulnesene	-	0.7	-	-
39	1508	1509	*E,E*-α-farnesene	0.2	-	-	-
40	1513	1514	*γ*-Cadinene	0.6	-	-	5.2
41	1522	1523	Lilial	1.8	1.1	-	-
42	1524	1522	*δ*-Cadinene	1.2	-	-	-
43	1576	1578	Spathulenol	1.8	1.9	-	3.5
44	1576	1579	Globulol	0.4	-	-	-
45	1590	1592	Viridiflorol	0.7	-	-	-
46	1611	1607	Tetradecanal	-	-	-	2.0
47	1800	1806	Nootkatone	0.2	-	-	-
Monoterpenes				11.0	1.5	2.8	3.2
Monoterpenoids				47.3	70.2	51.6	55.7
Sesquiterpenes				17.2	10.8	7.4	17.4
Sesquiterpenoids				3.1	1.9	-	3.5
Saturated hydrocarbons				-	-	14.1	2.6
Aromatics				0.1	0.5	21.6	2.6
Others				5.2	5.3	-	8.5
Total % of identified compounds				83.9	90.2	97.5	93.5

KI^a^: Kovat indices for the capillary column of HP-5MS; KI^b^: Kovat indices from the literature [20,21,22,23].

## Data Availability

Data presented in this study is contained within the article. Further inquiries can be directed to the corresponding author.
